# MicroRNA Profiling of Highly Enriched Human Corneal Epithelial Stem Cells by Small RNA Sequencing

**DOI:** 10.1038/s41598-020-64273-0

**Published:** 2020-05-04

**Authors:** Lavanya Kalaimani, Bharanidharan Devarajan, Umadevi Subramanian, Vanniarajan Ayyasamy, Venkatesh Prajna Namperumalsamy, Muthukkaruppan Veerappan, Gowri Priya Chidambaranathan

**Affiliations:** 1 Department of Immunology and Stem Cell Biology, Aravind Medical Research Foundation, Madurai, Tamil Nadu India; 2 Department of Biotechnology, Aravind Medical Research Foundation -Affiliated to Alagappa University, Karaikudi, Tamil Nadu India; 3 Department of Bioinformatics, Aravind Medical Research Foundation, Madurai, Tamil Nadu India; 4 Department of Molecular Genetics, Aravind Medical Research Foundation, Madurai, Tamil Nadu India; 5 Cornea Clinic, Aravind Eye Hospital and Postgraduate Institute of Ophthalmology, Madurai, Tamil Nadu India

**Keywords:** Adult stem cells, Non-coding RNAs

## Abstract

The objective of the study was to elucidate the microRNA (miRNA) profile of an enriched human corneal epithelial stem cell (CESC) population in comparison to differentiated central corneal epithelial cells (CCECs) by small RNA sequencing. The CESCs were enriched by differential enzymatic treatment to isolate the basal limbal epithelial cells followed by laser capture microdissection of cells with nucleus to cytoplasm ratio ≥0.7, from donor tissues. Small RNA sequencing was carried out using Illumina NextSeq. 500 platform and the validation of differentially expressed miRNAs by quantitative real-time PCR (qPCR) and locked nucleic acid miRNA *in-situ* hybridization (LNA-ISH). The sequencing identified 62 miRNAs in CESCs and 611 in CCECs. Six miRNAs: hsa-miR-21-5p, 3168, 143-3p, 10a-5p, 150-5p and 1910-5p were found to be significantly upregulated in enriched CESCs, which was further confirmed by qPCR and LNA-ISH. The expression of hsa-miR-143-3p was exclusive to clusters of limbal basal epithelial cells. The targets of the upregulated miRNAs were predicted to be associated with signaling pathways -Wnt, PI3K-AKT, MAPK and pathways that regulate pluripotency of stem cells, cell migration, growth and proliferation. Further studies are essential to elucidate their functional role in maintenance of stemness. The findings of the study also hypothesize the inherent potential of hsa-miR-143-3p to serve as a biomarker for identifying CESCs.

## Introduction

The anterior surface of the human eye is defined by the tear film and cornea. The cornea acts as the natural barrier by preventing the underlying delicate structures from harmful radiation and potent infections^1^. The terminally differentiated cells in the superficial layer of the human corneal epithelium are normally shed and the cell loss is replenished by the progeny of the corneal epithelial stem cells (CESCs) that reside in the basal epithelial layer of the limbus to maintain tissue homeostasis. The CESCs give rise to cells that can proliferate, differentiate and migrate centripetally^2,3^. These adult stem cells possess slow cycling^4^, label retaining cell property^5^ and has the ability to form holoclones^6,7^. Loss or dysfunction of the CESCs results in conjunctivalization, vascularization and loss of vision, a condition termed as limbal stem cell deficiency^8,9^.

Even though it is well accepted that the limbus is the site of stem cells for corneal epithelial homeostasis^10–12^, the regulatory mechanism governing the maintenance of these CESCs is still not clear. MicroRNAs (miRNAs) are non-coding RNAs approximately 22-nucleotide-long and they act as key players in regulating gene expression by RNAi machinery. In the last decade, the importance of miRNAs as a potential epigenetic regulator of stem cell potency, proliferation, differentiation and survival in embryonic stem cells^13,14^, induced pluripotent stem cells^15,16^ and adult tissue resident stem cells like human skin/hair follicle^17^ has been reported.

It has been demonstrated that only 25% of the isolated limbal basal epithelial cells are stem cells based on the analysis of two parameters, high levels of p63 expression and greater nucleus to cytoplasmic area^7^. It is crucial to make use of a highly enriched CESCs in order to identify the profile of miRNA specific to adult tissue resident stem cells. Therefore, in this study we have made use of the strategy we have developed earlier^7,18^ to enrich CESCs to the extent of 80% and this enriched population are known to express stem cell markers ΔNp63α and ABCG2^18^. It was thus possible to identify a specific profile of miRNA, significantly up-regulated in CESCs compared to differentiated CCECs^19^. Accordingly, the major findings of the study will serve as a platform to decipher the unresolved questions relating to the miRNA regulation of CESCs.

## Methods

### Samples

Human donor tissues were handled in accordance with the tenets of the Declaration of Helsinki and the study was approved by Institutional Ethics Committee Aravind Medical Research Foundation (RES2013038BAS). Informed consent was obtained for all donor eyes including the minors from the legally authorized representative - either the donor’s parents or family through the Eye Banks of Aravind Eye Care System (Madurai, Coimbatore, Pondicherry and Tirunelveli). The donor globes not suitable for transplantation (procured within 8 hours of death and received within 24 hours for research) and limbal rims obtained after corneal transplantation (received within 10 days of storage in Cornisol media at 4 °C in sterile condition) were included in this study. Inclusion criteria were donor age less than 70 years (range: 11 to 70 years), non-vascularized, with no history of diabetes and ocular infection. The donor globes were observed under stereo binocular microscope and those with intact limbus with radial ridges of palisades of Vogt were used for the study.

### Enrichment of corneal epithelial stem cells

The corneal region together with adjacent sclera was dissected out from the donor globes and the corneal endothelium was removed using a sterile cotton tip^20,21^. Central cornea was punched out using 8 mm trephine and the central corneal epithelial cells (CCECs) were isolated following the protocol of Arpitha *et al*.^20^. Briefly, the corneal epithelial sheet was separated by dispase II (2 mg/ml) treatment and the individual cells were obtained after 0.25% trypsin treatment. For enrichment of the CESCs, a two step-protocol was followed^18^. The limbal basal epithelial cells (LBECs) were isolated from limbal tissues by differential enzymatic treatment – 0.25% trypsin followed by 2 mg/ml dispase II treatment^7^. The cytosmears of isolated LBECs on membrane slide were stained with Giemsa. The cells were focused with 20X magnification and the image was projected on the computer monitor connected to an automated microscope. The area of nucleus and cytoplasm of each cell and the nucleus to cytoplasmic (N/C) ratio was then calculated using PALM Robo Software 4.3 SP2. The corneal epithelial stem cells (CESCs) with N/C ratio >0.7 were selectively marked and cut using Positioning and Ablation Laser MicroBeam (PALM) and collected on the adhesive cap of the collection tube which positioned to overhang the area under focus. Catapulted cells collected in the adhesive cap can be checked by moving it directly over the objective lens at any point of time during the collection. The enriched CESCs in the adhesive cap of the collection tube were then incubated for 30 minutes at 42 °C in 15 µL of extraction buffer (ARCTURUS PicoPure RNA Isolation Kit) and collected to the bottom of the tube from the cap by a brief centrifugation. The collected cell lysate was stored at −80 °C until RNA isolation^18,22^.

### Small RNA sequencing and data analysis

The total RNA was extracted from pooled CESCs (2046 cells) and CCECs (8.55 × 10^5^ cells) using ARCTURUS PicoPure RNA Isolation Kit from two donor tissues (Age: 60 and 62 years) for small RNA sequencing. Total RNA was used for small RNA library construction using TruSeq Small RNA Library Prep Kit (Illumina, California, USA) following manufacturer’s protocol. Libraries were quantified using Qubit dsDNA HS Assay Kit (Invitrogen, California, USA) and validated in bioanalyzer using High Sensitivity DNA Kit (Agilent, California, USA). Based on the library QC report, the library generated was suitable for sequencing on Illumina. For CCECs, library distribution was in the range of 120bp-180bp and for CESCs the range was 120bp-160bp indicating the presence of small RNA inserts. The effective insert length was in the range of ~10bp- 40 bp; with combined size of adapters flanking the library being ~120 bp. Cluster generation and sequencing was carried out using NextSeq. 500 High-Output v2 Kit (Illumina, Inc.) using 75 cycles chemistry at the Genotypic Technology Pvt Ltd (Bangalore, India).

First, the quality assessment of raw deep sequencing data was performed with the FastQC tool. Adapter and low-quality reads were discarded from raw sequencing data using our Perl script, allowing no mismatches for adapter identification. Further, the data were uploaded into Oasis 2.0 online software^23,24^ for the miRNA analysis. Briefly, the data with the read size (15–32 nt) and low abundance reads (<5 reads) were discarded and remaining data were aligned to *Homo sapiens* hg19 genome reference. For differential expression (DE) analysis, NOISeq R software package were applied on the TMM normalized reads (R version 3.5.1). The variable expression of miRNAs between two cell types was considered significant when the fold change was 2 or greater and the probability score was more than 0.9. The results were represented in the volcano plot constructed by R version 3.5.1^25^ based on the NOISeq scores: the M-value and D-value.

### Quantitative real time PCR

For confirmation of the sequencing data, quantitative real time PCR was carried out using (i) CESCs from 33 pairs of limbal rims (cells from 11 pairs were pooled as one sample to get around 3500 cells/pool, n = 3) and CCECs from three pairs of corneal button and (ii) LBECs and CCECs from limbal rims and corneal buttons respectively, were pooled to obtain a minimum of 1 × 10^6^ cells each (pool 1: 7 pairs; pool 2: 7 pairs; pool 3: 6 pairs of tissues), but after ensuring that there was no bias of age. The RNA concentration was estimated using Qubit RNA HS Assay Kit (Invitrogen, California, USA) in Qubit2.0. fluorometer. RNA (15 ng) from CESCs (n = 3) and CCECs (n = 3) and 1 µg of RNA from LBECs (n = 3) and CCECs (n = 3) was reverse transcribed using miScript II RT Kit (Qiagen, Hilden, Germany) according to manufacturer’s protocol. Due to the low quantity of RNA (15 ng) obtained, the reverse transcribed cDNA from CESCs was pre-amplified using miScript PreAMP PCR Kit (Qiagen, Hilden, Germany) for 12 cycles (Denaturation: 30 seconds at 94 °C and annealing/ extension: 3 minutes at 60 °C) preceded by initial activation: 15 minutes at 95 °C. Hence the same protocol was followed for CCECs for comparison. The qPCR amplification was performed for 40 cycles using 2X miScript SYBR Green PCR Master Mix, 10X miScript Universal Primer and miRNA specific miScript Primer Assays (Supplementary Table S1 for list of miRNA Primer Assays used and Table S2 for custom designed miRNA primer sequence). Experiment was done in triplicates and signals were normalized to small nucleolar RNA U6 (RNU6B) which was run in parallel as reference miRNA. Relative miRNA expression was calculated using comparative threshold cycle (Ct) method (2^-ΔΔCt^) and the result was represented as mean ± SEM. Statistical analysis of qPCR data was carried out using Mann-Whitney U test and miRNAs with fold change >2 and p value <0.05 were considered significant.

### LNA *in-situ* hybridization

For validation of differential expression of miRNAs in its native environment, LNA *in-situ* hybridization was carried out following protocol of Obernosterer *et al*.^26^ with some modifications. Briefly, The corneoscleral region from the donor eye ball was separated and fixed with 4% paraformaldehyde (Sigma-Aldrich, Missouri, United States) for 30 minutes at room temperature and then the tissue was incubated overnight at 4 °C in 30% sucrose solution prepared in 1X phosphate buffered saline (PBS) of pH 7.4 (Ambion, California, United States). The tissue was embedded in optimal cutting temperature (OCT) compound (Leica, Wetzlar, Germany), and cryosections (10 μm) of limbal epithelium and corneal epithelium were taken on SuperFrost Plus slides (Thermofisher Scientific, Massachusetts, United States). The cryosections were fixed with ice cold acetone for 10 minutes at room temperature and then hybridized with biotinylated LNA miRNA probes (Eurogentec, Liège, Belgium; Supplementary Table S3 for list of LNA miRNA probes used) at 50 °C overnight by placing coverslip over the sections to prevent drying. U6 small nucleolar RNA probe and LNA scrambled microRNA probe were used as positive and negative control respectively. After hybridization, the slides were soaked in prewarmed 5X SSC Buffer (Ambion, California, United States) to remove the coverslips. The sections were incubated at 60 °C for 1 hour in 0.2X SSC buffer followed by blocking with 10% fetal bovine serum (Invitrogen, California, USA) in 0.1 M Tris (pH 7.5) and 0.15 M sodium chloride solution at room temperature for 1 hour. To detect the hybridized probes, the sections were incubated with Streptavidin-Fluorescein Isothiocyanate (FITC) Conjugate (BD Biosciences, New Jersey, United States) in 1:1000 in 5% bovine serum albumin (Sigma-Aldrich, Missouri, United States) at 25 °C in dark for 2 hours. The sections were then washed twice with 1X PBS for 5 minutes and mounted using fluorescent mounting media with propidium iodide (Vector Laboratories, California, United States). The images were acquired using confocal laser scanning microscope (Leica SP8, Germany).

### miRNA Target prediction, Gene Ontology and Pathway Analysis

miRWalk (Version 3.0) and mirDIP (Version 4.1.1.6) were used to predict the target genes of miRNAs. Experimentally validated targets from miRWalk and which were targets with> 10 sources in mirDIP databases were filtered for the analysis. For pathway prediction, ClueGO package in Cytoscape 3.2.0 was used. KEGG pathway list was chosen with p-value ≤ 0.01. Further, the network of miRNAs, target genes and the associated pathways was constructed by Cytoscape 3.2.0^27^.

### Statistical analysis

Mann-Whitney U test was performed to determine the statistical significance between the two experimental groups and p < 0.05 was considered statistically significant. STATA 14.0 (Texas, USA) statistical software was used for analysis. Non-parametric test, Mann-Whitney U was chosen since the qPCR data followed non-gaussian distribution based on the Shapiro-Wilk normality test.

## Results

### Characteristics of small RNA sequencing and differentially expressed miRNAs between CESCs and CCECs

**Figure 1:**
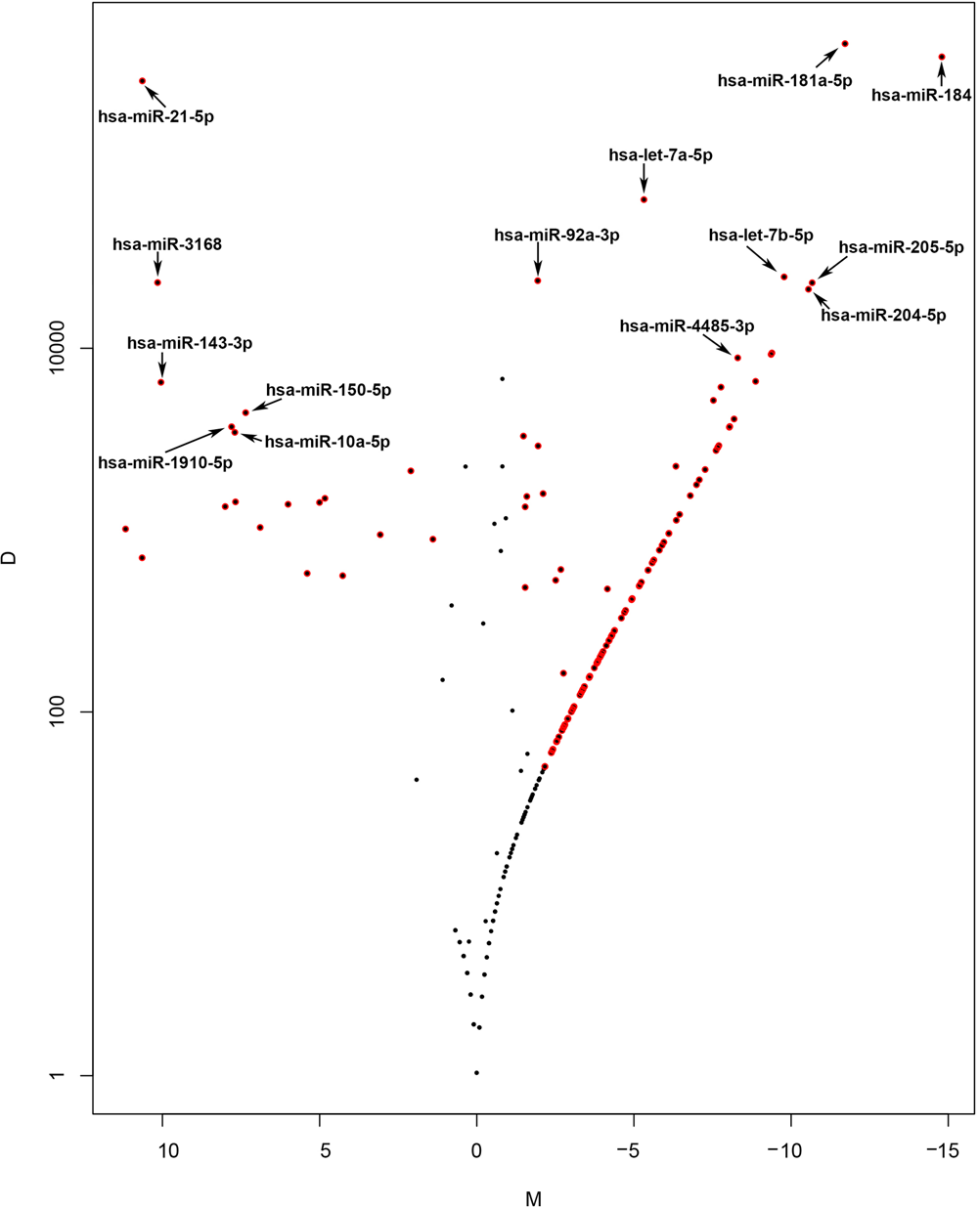
Volcano plot showing differentially expressed miRNAs in CESCs and CCECs. M-D values in noise are represented by black dots, and miRNAs with p-value ≤ 0.9 by red dots. M –value (Mean fold change): log2(x1/x2). D –value (Difference in expression levels): |x1-x2 | . where x1 represents the expression level in CESCs and x2 in CCECs.

**Figure 2:**
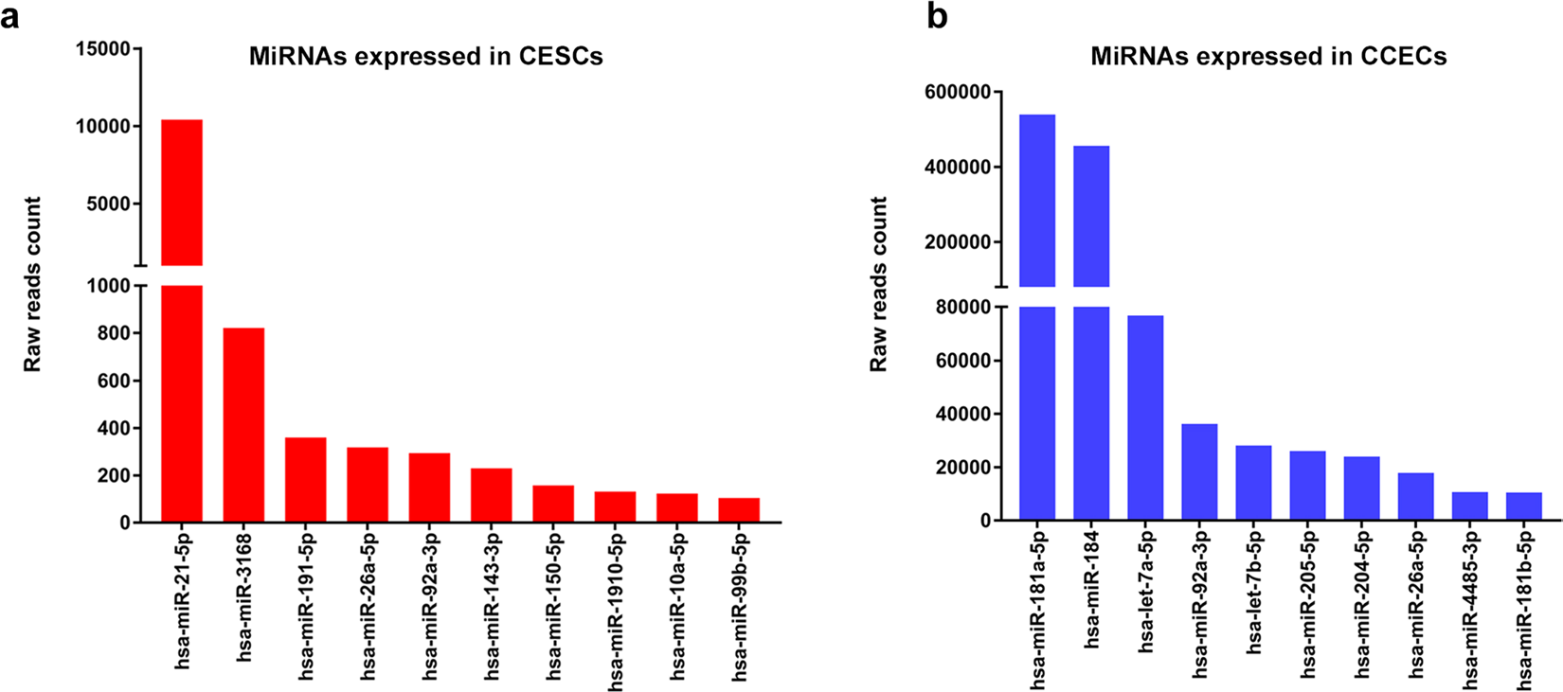
MicroRNAs with high read counts in corneal epithelial stem cells and central corneal epithelial cells by small RNA sequencing. (**a**) Bar diagram showing the top ten miRNAs expressed in enriched CESCs based on raw read counts. (**b**) Bar diagram showing the top ten miRNAs expressed in CCECs based on raw read counts.

The entire quantity of total RNA isolated from enriched CESCs (pooled cells from two pairs of donor tissues) was used for library preparation as the concentration was lesser than the detection limit in the Qubit analysis. For CCECs, 10 ng of total RNA (RIN 2.8) was used. The sequencing generated more than 11 million reads in CESCs and CCECs. After quality assessment and refinement, the high quality reads were mapped to human hg 19 genome. A total of 38 miRNAs in CESCs and 301 miRNAs in CCECs were detected. Further, differential expression (DE) analysis using NOISeq after normalization were performed on all detected miRNAs. A total of 127 DE miRNAs were identified in CESCs compared to CCECs with fold change >±2. Among them 29 miRNAs were upregulated and 64 miRNAs were downregulated. The fold change values of the miRNAs were summarized as volcano plot in Fig. 1. The top ten miRNAs that were highly expressed in CESCs (Fig. 2a) and CCECs (Fig. 2b) based on raw reads counts were represented graphically. Based on fold difference between CESCs and CCECs and raw reads counts, fourteen differentially expressed miRNAs and two miRNAs without significant difference in expression were selected for validation by qPCR expression analysis (Table 1).

**Table 1:** Differentially expressed miRNAs CESCs vs. CCECs validated by qPCR.

miRNA	NGS fold change	qPCR fold change
hsa-miR-21-5p	11	7.34 ± 0.38
hsa-miR-3168	10	3.71 ± 0.72
hsa-miR-143-3p	10	76.44 ± 3.07
hsa-miR-150-5p	7	13.86 ± 1.49
hsa-miR-1910-5p	8	7.76 ± 0.17
hsa-miR-10a-5p	8	11.76 ± 0.75
hsa-miR-181a-5p	−12	−894 ± 83.22
hsa-miR-184	−15	−2409 ± 214.9
hsa-let-7a-5p	−5	−1.66 ± 0.062
hsa-let-7b-5p	−10	−1.98 ± 0.11
hsa-miR-205-5p	−11	−14.64 ± 0.39
hsa-miR-204-5p	−11	−2.18 ± 0.08
hsa-miR-4485-3p	−9	−10.63 ± 1.21
hsa-miR-92a-3p	−2	−5.47 ± 0.16
hsa-miR-191-5p	NS	1.10 ± 0.02
hsa-miR-26a-5p	NS	1.99 ± 0.11

NS-not significant.

### Validation of differentially expressed miRNAs by qPCR

**Figure 3:**
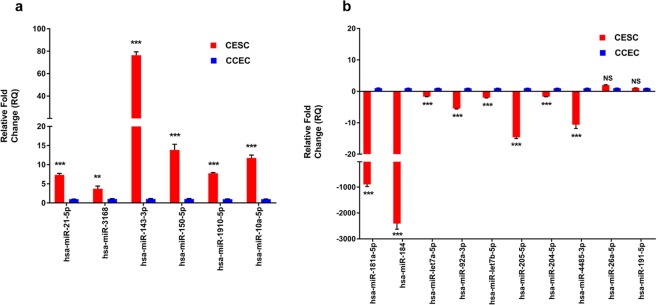
Quantitative Real time PCR validation of differentially expressed miRNAs. Relative miRNA expression (RQ) in (**a**) miRNAs highly expressed in CESCs in comparison to CCECs and (**b**) miRNAs highly expressed in CCECs in comparison to CESCs by qPCR using SYBR Green chemistry. Each sample (n = 3) was run in triplicate. The data were expressed as mean ± SEM and relative fold change of expression (RQ) was calculated by 2^-∆∆CT^ method after normalization with RNU6B (Reference microRNA). (**P < 0.01; ***P < 0.001; NS *P* > 0.05; Mann–Whitney U test).

Validation of the sequencing data was carried out using RNA extracted from enriched CESCs and CCECs (after pooling cells from different donors) as specified under materials and methods. All six miRNAs hsa-miR-3168, hsa-miR-21-5p, hsa-miR-143-3p, hsa-miR-150-5p, hsa-miR-1910-5p and hsa-miR-10a-5p showed higher expression in CESCs with significant fold change (>2) compared to CCECs confirming the sequence data. Among them the magnitude of fold difference was much higher for hsa-miR-143-3p with 76.44 ± 3.07, while a ten-fold change was observed for hsa-miR-10a-5p and hsa-miR-150-5p and a five-fold change was observed for hsa-miR-21-5p and hsa-miR-1910-5p (Fig. 3a). The nine miRNAs that are identified to be down regulated in CESCs by sequencing, hsa-miR-184, hsa-miR-181a-5p, hsa-miR-92a-3p, hsa-miR-4485-3p, hsa-miR-205-5p, hsa-miR-99b-5p, hsa-miR-204-5p, hsa-let7a-5p and hsa-let7b-5p had reduced expression in CESCs compared to CCECs, thus validating the sequencing data. The top two miRNAs expressed in CCECs had fold change of 2409 ± 214.9 (hsa-miR-184) and 894 ± 83.22 (hsa-miR-181a-5p) compared to that of CESCs. Hsa-miR-26a-5p and hsa-miR-191-5p did not show significant difference in the expression pattern between the two populations (Fig. 3b).

### Localization of differentially expressed miRNAs by LNA *in-situ* hybridization

Expression of miRNAs hsa-miR-21-5p, hsa-miR-3168, hsa-miR-10a-5p, hsa-miR-1910-5p, hsa-miR-150-5p, hsa-miR-143-3p and hsa-miR-26a-5p was analyzed in cryosections of limbal epithelium (LE) and corneal epithelium (CE).

**Figure 4:**
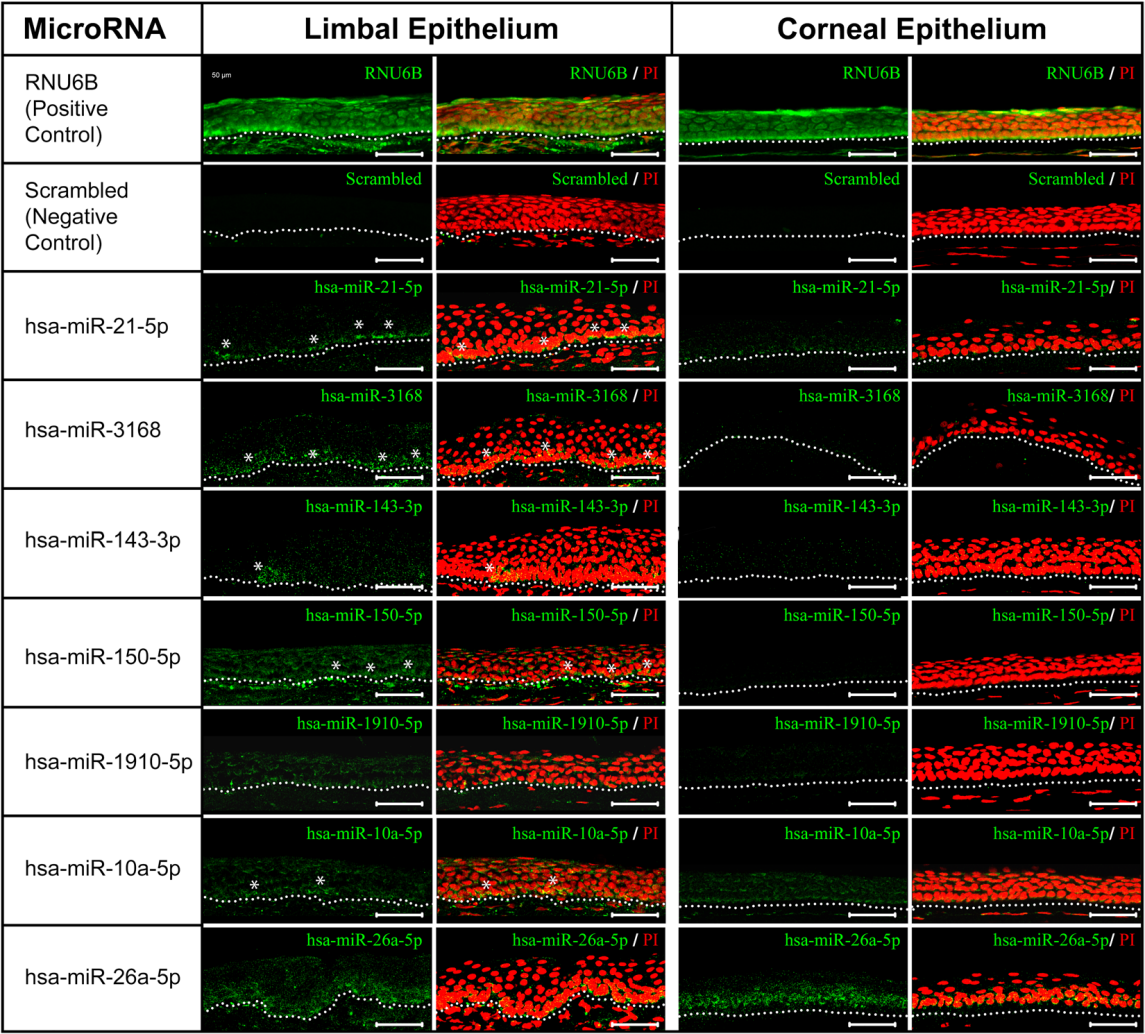
Locked nucleic acid *in-situ* hybridization of miRNAs highly expressed in CESCs. Expression of hsa-miR-21-5p, hsa-miR-3168 and hsa-miR-10a-5p (green) was higher in clusters of limbal basal epithelial cells compared to corneal epithelial cells, while the expression of hsa-miR-143–3p was restricted to clusters of cells in the limbal basal epithelium. Expression of hsa-miR-150-5p was evident in all layers of limbal epithelium however highly prominent in a few clusters in basal layer. Hsa-miR-1910-5p had discontinuous positivity in limbal basal epithelial layer, while hsa-miR-26a-5p had equal expression in the basal layer both limbal and corneal epithelium. Nuclei were stained with propidium iodide (PI, red). Positive control RNU6B was detected in all layers of epithelium both in limbus and cornea, whereas no signal was detected when hybridized with scrambled sequence. Asterisks represent the positivity in clusters. The dotted line demarcates the termination of the epithelium and beginning of the underlying stroma. Scale bar: 50 µm.

The differentially expressed miRNAs, hsa-miR-21-5p, hsa-miR-3168, hsa-miR-150-5p, hsa-miR-143-3p, hsa-miR-1910-5p and hsa-miR-10a-5p had higher expression in limbal basal epithelial cells compared to that of corneal basal epithelial cells. Interestingly the expression of hsa-miR-143-3p was exclusive to the clusters of cells in limbal basal epithelium. Among the limbal basal epithelial cells, higher expression of hsa-miR-21-5p, hsa-miR-3168, hsa-miR-150-5p and hsa-miR-10a-5p was observed in a few clusters, while a discontinuous positivity was observed for hsa-miR-1910-5p. The miRNA hsa-miR-26a-5p had equal expression in both limbal and corneal epithelial basal cells (Fig. 4).

### Pathway and Gene Ontology analysis

**Figure 5:**
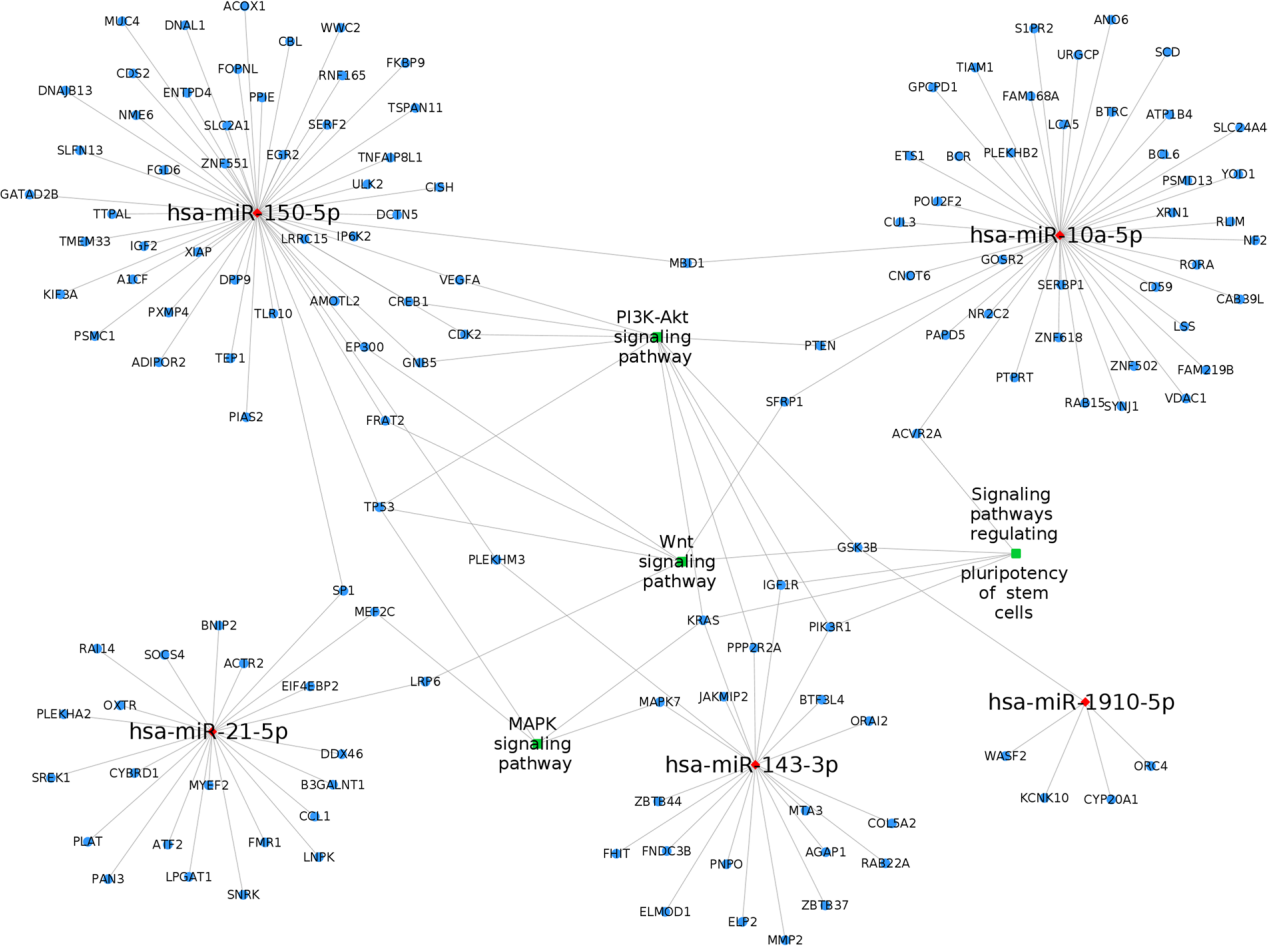
Network of differentially expressed miRNAs between CESCs and CCECs, their target genes and the stemness related pathways associated with them. Nodes red in colour are the miRNAs, blue are genes and green are their associated pathways.

For the functional analysis, the targets of six DE miRNAs hsa-miR-3168, hsa-miR-10a-5p, hsa-miR-143-3p, hsa-miR-150-5p, hsa-miR-1910-5p, and hsa-miR-21-5p were predicted and their associated pathways and gene ontology were analyzed (Fig. S3). A total of 1055 targets were predicted for six miRNAs and they were enriched into 55 pathways with FDR < 0.01, are shown in Table S4. Further, stemness related pathways were visualized (Fig. 5). The target genes *GSK3B* (regulated by hsa-miR-1910-5p), *KRAS* (hsa-miR-143-3p), and *PIK3R1* (hsa-miR-143-3p) were associated with PI3K-Akt signaling pathway and signaling pathways regulating pluripotency of stem cells. In addition, *GSK3B* was associated with Wnt signaling pathway, and KRAS with MAPK signaling pathway. Particularly in signaling pathway regulating the pluripotency of stem cells, hsa-miR-143-3p dysregulate the pathway through the target genes *IGF1R, PIK3R1, KRAS*; hsa-miR-1910-5p by *GSK3B*, and *ACVR2A* by hsa-miR-10a-5p.

## Discussion

MicroRNA profiling of total human limbal epithelium^28^, human limbal basal epithelium^29^ and mouse limbal basal epithelium^30,31^ in comparison to central corneal epithelium revealed many differentially expressed miRNAs. In addition, reports are available on the role of miRNAs in differentiation^28,32^, transdifferentiation^33^, angiogenesis^34–36^, wound healing^37,38^, corneal transplant rejection^39^, corneal neovascularization^40^ and ocular infections^41^. But only a few reports are available on association of miRNAs in the regulation of stemness. The major challenge of profiling the miRNAs in CESCs was the low stem cell content (3-5%) in the total limbal epithelium. Hence an attempt was made to profile the miRNAs specific to adult stem cells of the limbus using a small number of highly enriched CESCs as close as eighty percent^18^ by small RNA sequencing platform. Since the yield of CESCs was minimum after enrichment, Arcturus picopure RNA isolation kit was used in this study for RNA isolation, to reduce the loss of short structured RNA with low GC during extraction from small number cells^42^.

Small RNA sequence data of CCECs was used as a quality control for the sequencing. Comparison of the data with published literature^29,43^ using total cornea or corneal epithelium (by NGS or microarray) identified a similar profile of miRNAs, thereby confirming the validity of the data. However, only 38 miRNAs were identified in CESC population and this could possibly due to the following concerns (i) low input RNA, that could not be quantified before library preparation ii) usage of LCM for enrichment process and iii) difference in method of isolation of CESCs and CCECs. In spite of this drawback, miRNAs identified to be highly expressed in the CESCs were confirmed to be specific by *in-situ* hybridization and qPCR.

Though the number of miRNAs detected in CESCs was only one tenth compared to CCECs, hsa-miR-3168, hsa-miR-21-5p, hsa-miR-143-3p, hsa-miR-150-5p, hsa-miR-1910-5p and hsa-miR-10a-5p had greater than seven folds increase in expression in the enriched CESCs indicating their specificity to stem cells. Among these six miRNAs, hsa-miR-3168^44^ and hsa-miR-1910-5p^45^ were reported as novel miRNAs in human embryonic stem cells. Understanding their functional role in regulating adult tissue resident stem cells will be of significance. The remaining four miRNAs were reported to have a role in regulating other stem cells. Hsa-miR-21-5p suppresses marrow derived endothelial progenitor cells proliferation by activating TGFβ signaling through down regulation of WW domain-containing protein 1 (WWP1)^46^. Similarly, miR-10a down regulates proliferation of human cardiomyocyte progenitor cells by targeting *GATA6*^47^. Hsa-miR-143-3p and hsa-miR-150-5p are known to be associated with self-renewal of mouse embryonic stem cells and human liver cancer stem cells respectively. MiR-143 promotes self-renewal by suppressing de novo methyltransferase gene *DNMT3A*^48^ and miR-150 inhibits cell proliferation by targeting *C-MYB*^49^.

Validation of the sequencing data by qPCR analysis revealed that fourteen differentially expressed miRNAs to have similar expression pattern (Fig. 3). In addition, all the differentially expressed miRNAs had several magnitudes of fold difference in CESCs than LBECs when compared to CCECs independently (Fig. S2). In addition, the up regulation of hsa-miR-3168 as well as the down regulation of hsa-let7a-5p and hsa-miR-204-5p was identified only with the enriched CESCs but not with LBECs. Thus, these observations highlight the importance of enrichment of CESCs and confirming the specificity of the identified miRNAs.

LNA *in-situ* hybridization revealed the location of the miRNAs in corneal and limbal tissue. Among the miRNAs that were highly expressed in CESCs, hsa-miR-3168, hsa-miR-21-5p, hsa-miR-150-5p and hsa-miR-1910-5p had higher expression in limbal basal epithelial layer compared to that of the corneal basal epithelial layer. Further, a few clusters of small cells had strong positivity (probably stem cells) compared to that of the other limbal basal epithelial cells. Likewise, the expression of hsa-miR-143-3p was confined only to clusters in limbal basal epithelial layer, indicating its very high specificity to stem cells.

The predicted targets of these miRNAs that were highly expressed in CESCs were identified to be associated with pathways regulating pluripotency of stem cells and other pathways including MAPK signaling, Ras signaling, Hippo signaling, ErbB signaling, Wnt signaling and PI3K-Akt signaling. Reports are available on the significance of Wnt^50^, PI3K/Akt^51^, MAPK^52^ signaling pathways in the regulation of stem cells. The direct targets of the miRNAs (highly expressed in CESCs) predicted to be involved in these signaling pathways are listed in Table 2. These targets are known to be associated with cell proliferation, differentiation, migration and apoptosis. Further studies are therefore essential to confirm the functional role of the identified miRNAs and their targets in the regulation of stemness in CESCs.

**Table 2:** List of direct targets of selected miRNAs highly expressed in CESCs and their functional role.

miRNA	Targets	Functional role	Reference
Hsa-miR-143-3p	*MAPK7*	Inhibits adipocyte proliferation and enhance their differentiation	^58^
		Inhibits proliferation, cell migration and invasion of breast cancer	^59^
	*KRAS*	Suppresses colorectal cancer cell growth	^60^
	*IGF1R*	Regulates cell proliferation and apoptosis	^61^
		Regulation of myogenesis	^62^
	*PIK3R1*	Down regulation promotes propagation, migration, epithelial mesenchymal transition and stem cell phenotype of renal canal cells	^63^
Hsa-miR-21-5p	*MEF2C*	Crucial for neuronal function	^64^
Hsa-miR-10a-5p	*PTEN*	Tumor suppressor	^65^
Hsa-miR-150-5p	*VEGFA*	Decrease migration, invasion and angiogenesis in Rheumatoid arthritis	^66^
	*CREB1*	A novel Wnt effector that may enhance epithelial mesenchymal transition of colorectal cancer cells	^67^
	*CDK2*	Inhibits proliferation and tumorigenicity via retarding G1/S phase transition	^68^
	*TP53*	Promotes proliferation of lung cancer cells	^69,70^

One of the limitations of this study is the use of donor tissues. The RNA expression could have been altered due to the associated ischemia^53^ and the additional processing of the cells for enrichment of stem cells^54^. In spite of the low input of RNA for sequencing and low number of miRNAs identified in this study, confirmation of the high expression of miRNAs hsa-miR-3168, hsa-miR-21-5p, hsa-miR-143-3p, hsa-miR-150-5p, hsa-miR-1910-5p and hsa-miR-10a-5p in CESCs indicates their significant role in the regulation of stem cells. Transfection studies using hsa-miR-143-3p and hsa-miR-150-5p mimics in the primary limbal epithelial cells (n = 3) identified their increased colony forming potential, specifically their ability to form holoclones (based on colony morphology as defined by Barrandon and Green^55^), thereby indicating a strong regulatory influence of these miRNAs on maintenance of stemness^56,57^. Thus, the data generated serves as a platform to study the miRNAs associated with the maintenance of stemness. Further studies are essential to elucidate the functional role of these miRNAs in regulating the stemness in CESCs and the associated signaling pathways.

## Supplementary information

Supplementary Information.
