# Supplementary material for: MicroRNA Profiling of Highly Enriched Human Corneal Epithelial Stem Cells by Small RNA Sequencing
Source: Sci Rep. 2020 May 4;10:7418. doi: 10.1038/s41598-020-64273-0 (PMC7198595; doi:10.1038/s41598-020-64273-0)
Supplement: Supplementary file 1 — Supplementary Information. [file 41598_2020_64273_MOESM1_ESM.docx]

**Supplementary information**

**Title:** MicroRNA Profiling of Highly Enriched Human Corneal Epithelial Stem Cells by Small RNA Sequencing

**Running title:** MicroRNA profiling of CESCs

**Authors:** Lavanya Kalaimani^1,2^, Bharanidharan Devarajan^3^, Umadevi Subramanian^3^, Vanniarajan Ayyasamy^4^, Venkatesh Prajna Namperumalsamy^5^, Muthukkaruppan Veerappan^1^, Gowri Priya Chidambaranathan ^1,2*^

**Affiliations:**

^1^ Department of Immunology and Stem Cell Biology, Aravind Medical Research Foundation, Madurai, Tamil Nadu, India.

^2^ Department of Biotechnology, Aravind Medical Research Foundation -Affiliated to Alagappa University, Karaikudi, Tamil Nadu, India.

^3^ Department of Bioinformatics, Aravind Medical Research Foundation, Madurai, Tamil Nadu, India.

^4^ Department of Molecular Genetics, Aravind Medical Research Foundation, Madurai, Tamil Nadu, India.

^5^ Cornea Clinic, Aravind Eye Hospital and Postgraduate Institute of Ophthalmology, Madurai, Tamil Nadu, India.

**Figure S1**

**
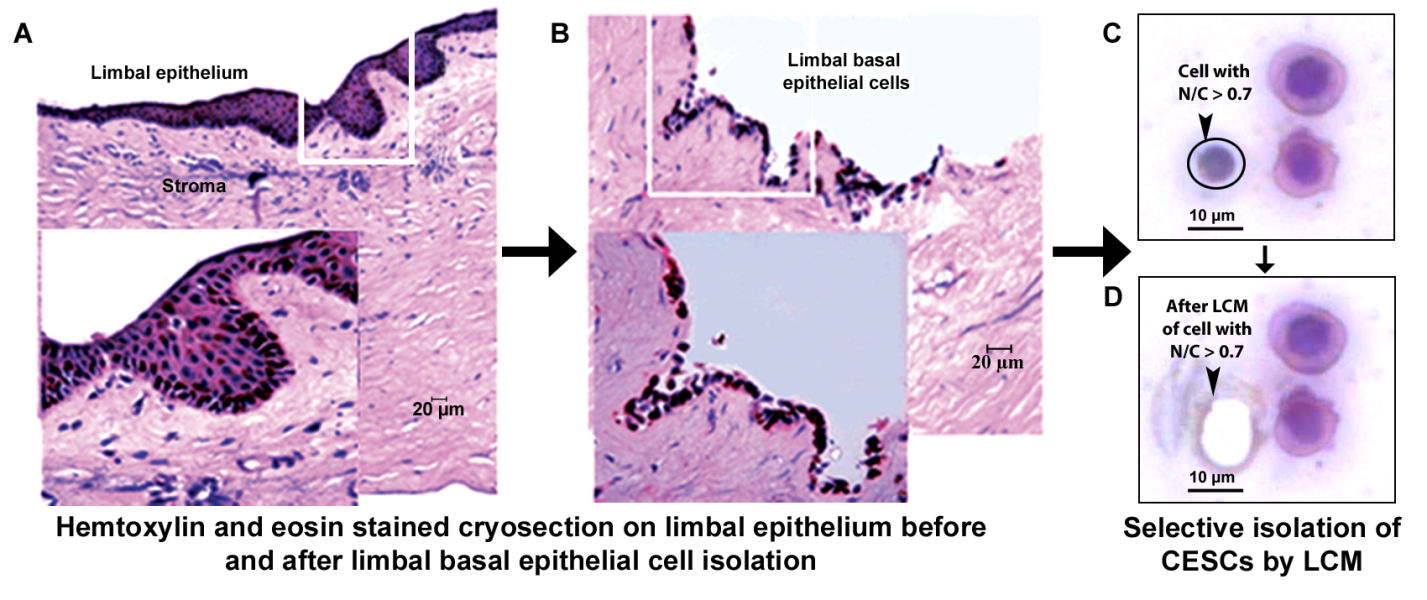
**

**Figure S1. Two step protocol for enrichment of CESCs**

Hematoxylin and eosin stained cryosection of limbal epithelium (a) before trypsin treatment (b) after trypsin treatment. Cytosmear of limbal basal epithelial cells on membrane slide (c) before and (d) after laser capture micro dissection of a cell with N/C ratio > 0.7 represented with arrows.

**Figure S2**


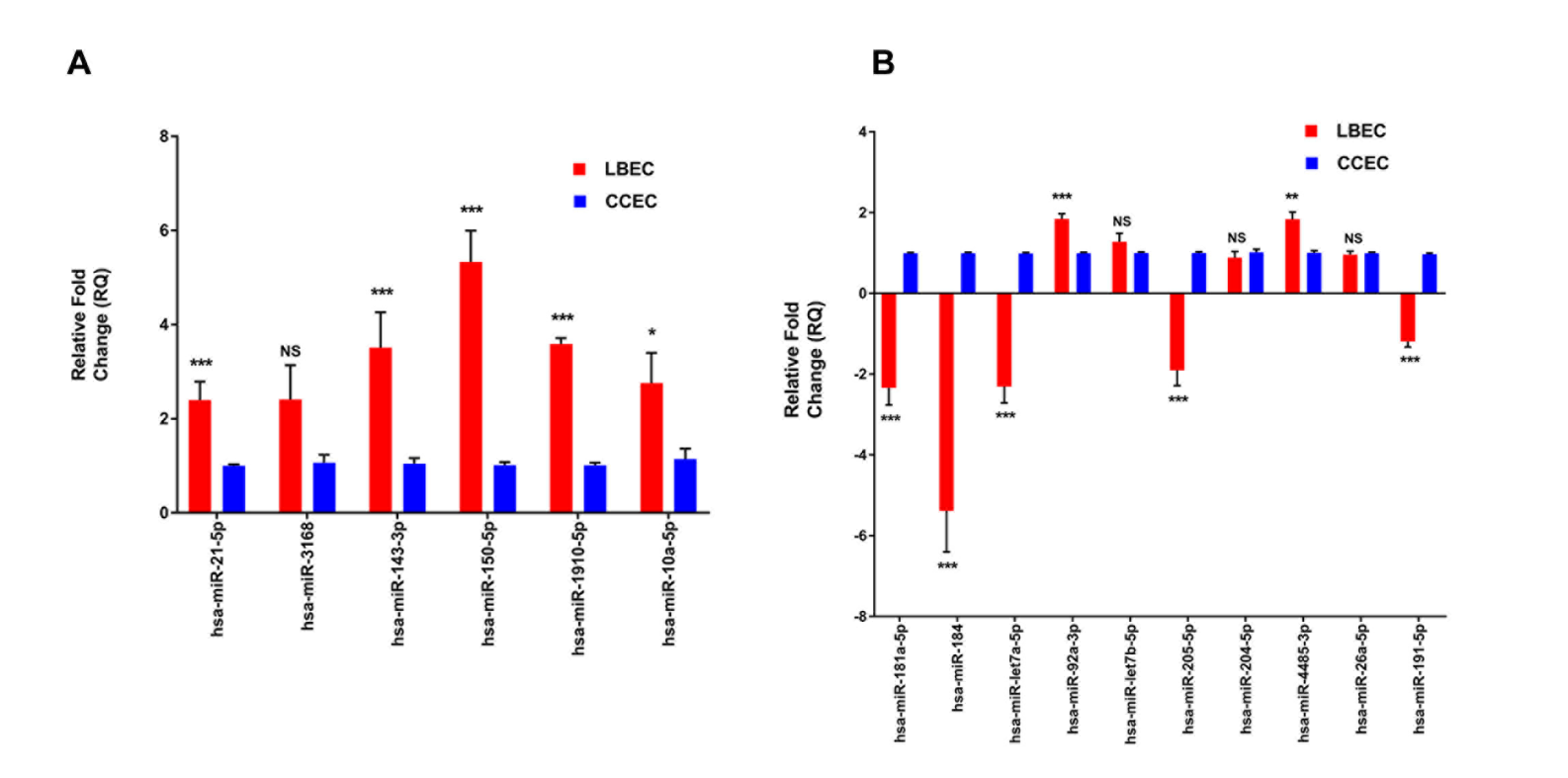


**Figure S2. Relative expression profile of miRNAs in LBECs vs. CCECs by qPCR**

Relative miRNA expression (RQ) in (A) miRNAs highly expressed in LBECs in comparison to CCECs and (B) miRNAs highly expressed in CCECs in comparison to LBECs by qPCR using SYBR Green chemistry. Each sample (n = 3) was run in triplicate. The data were expressed as mean ± SEM and relative fold change of expression (RQ) was calculated by 2^-∆∆CT^ method after normalization with RNU6B (reference microRNA).

(**P* < 0.05; ***P* < 0.01; ****P* < 0.001; NS *P* > 0.05; Mann–Whitney U test).

**Figure S3**


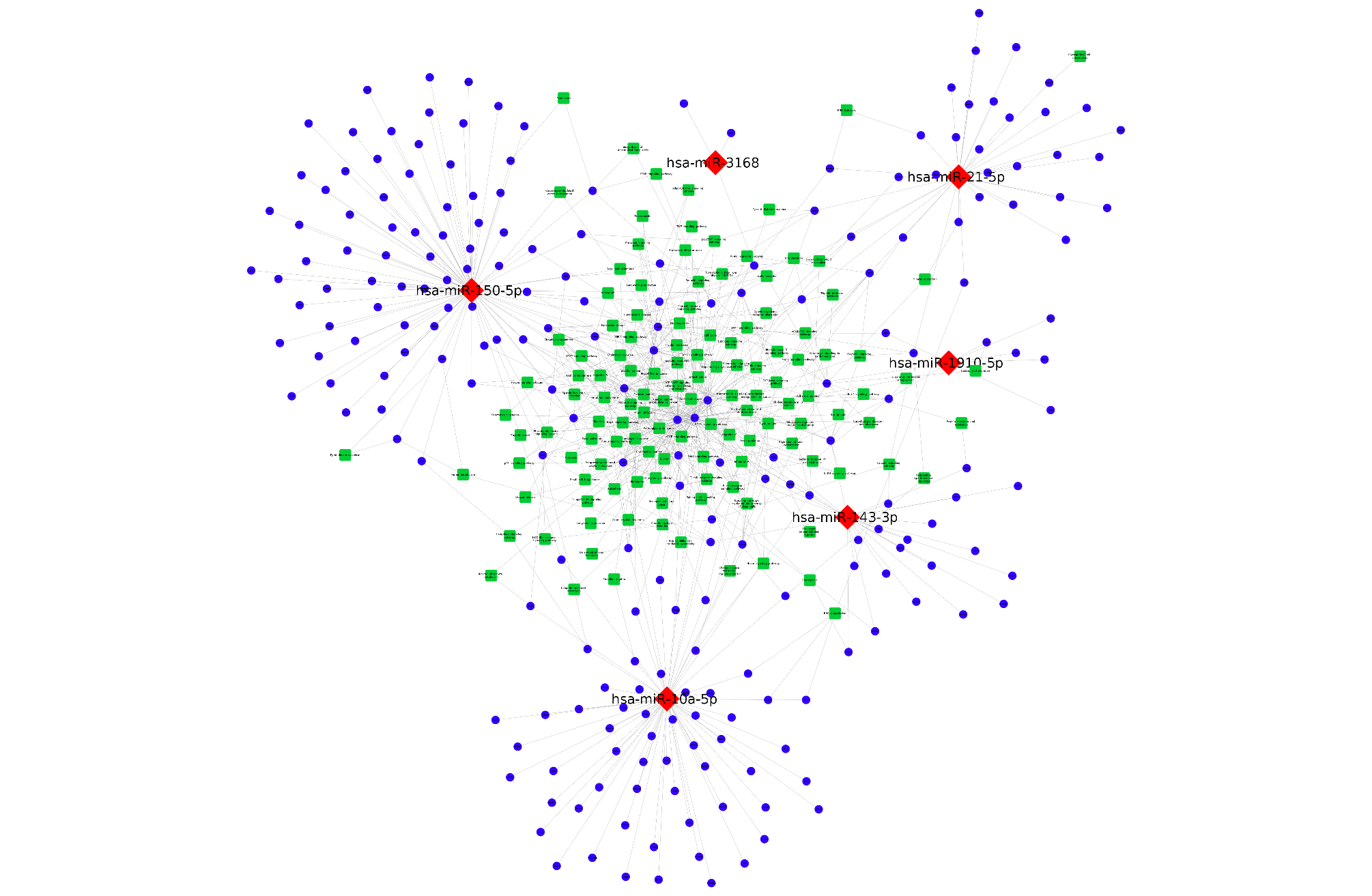


**Figure S3: Network of qPCR validated differentially expressed miRNAs between CESCs and CCECs, their target genes and pathways associated with them**

Nodes red in colour are the miRNAs, blue are genes and green are their associated pathways.

**Table S1: List of miRNA primer assays**

| **S.No** | **miRNAs** | **Primer assay** | **Catalogue (Qiagen)** |
| --- | --- | --- | --- |
| 1 | hsa-miR-21-5p | Hs_miR-21_2 | MS00009079 |
| 2 | hsa-miR-3168 | Hs_miR-3168_1 | MS00020804 |
| 3 | hsa-miR-191-5p | Hs_miR-191_1 | MS00003682 |
| 4 | hsa-miR-26a-5p | Hs_miR-26a_2 | MS00029239 |
| 5 | hsa-miR-92a-3p | Hs_miR-92_1 | MS00006594 |
| 6 | hsa-miR-143-3p | Hs_miR-143_1 | MS00003514 |
| 7 | hsa-miR-150-5p | Hs_miR-150_1 | MS00003577 |
| 8 | hsa-miR-1910-5p | Hs_miR-1910_1 | MS00016464 |
| 9 | hsa-miR-10a-5p | Hs_miR-10a_2 | MS00031262 |
| 10 | hsa-miR-181a-5p | Hs_miR-181a_2 | MS00008827 |
| 11 | hsa-miR-184 | Hs_miR-184_1 | MS00003640 |
| 12 | hsa-let-7a-5p | Hs_let-7a_2 | MS00031220 |
| 13 | hsa-miR-205-5p | Hs_miR-205_1 | MS00003780 |
| 14 | hsa-let-7b-5p | Hs_let-7b_1 | MS00003122 |
| 15 | RNU6B | Hs_RNU6B_13 | MS00014000 |

**Table S2: Custom designed miRNA primer sequence**

| S. No. | miRNAs | Primer sequence (5’→3’) (Forward primer) |
| --- | --- | --- |
| 1 | hsa-miR-204-5p | TTCCCTTTGTCATCCTAT |
| 2 | hsa-miR-4485-3p | TAACGGCCGCGGTACCCT |

Universal reverse primers for miRNA PCR amplification was provided in miRScript SYBR Green PCR Kit (Qiagen)

**Table S3: Information of locked nucleic acid *in-situ* hybridization miRNA detection probes**

| **MicroRNA** | **Target species** | **Probe Sequence** |
| --- | --- | --- |
| hsa-miR-21-5p | Human | TCAACATCAGTCTGATAAGCTA |
| hsa-miR-26a-5p | Human | AGCCTATCCTGGATTACTTGAA |
| hsa-miR-143-3p | Human | AGCTACAGTGCTTCATCTCA |
| hsa-miR-3168 | Human | GTCTGACTGTAGAACTC |
| hsa-miR-150-5p | Human | CACTGGTACAAGGGTTGGGAGA |
| hsa-miR-1910-5p | Human | AGGCGGCAGGCACAGGACT |
| hsa-miR-10a-5p | Human | CACAAATTCGGATCTACAGGGTA |
| LNA Scrambled -miR | Human/ mouse | GTGTAACACGTCTATACGCCCA |
| U6 snRNA | Human/ mouse | CACGAATTTGCGTGTCATCCTT |

**Table S4: Differentially expressed miRNAs target associated pathways by GO analysis**

| **UNIQUE_ID** | **KEGG Pathways** | **p-value** | **FDR** |
| --- | --- | --- | --- |
| KEGG:04740 | Olfactory transduction | 5.1790E-38 | 1.38E-35 |
| KEGG:05200 | Pathways in cancer | 1.7361E-17 | 4.60E-15 |
| KEGG:05205 | Proteoglycans in cancer | 3.9623E-17 | 1.05E-14 |
| KEGG:04360 | Axon guidance | 3.4427E-14 | 9.05E-12 |
| KEGG:03010 | Ribosome | 3.9085E-13 | 1.02E-10 |
| KEGG:04722 | Neurotrophin signaling pathway | 7.7612E-13 | 2.03E-10 |
| KEGG:04010 | MAPK signaling pathway | 4.6782E-12 | 1.22E-09 |
| KEGG:04144 | Endocytosis | 4.7004E-12 | 1.22E-09 |
| KEGG:04015 | Rap1 signaling pathway | 2.0834E-11 | 5.38E-09 |
| KEGG:00190 | Oxidative phosphorylation | 1.0691E-09 | 2.75E-07 |
| KEGG:04510 | Focal adhesion | 3.4613E-09 | 8.86E-07 |
| KEGG:04014 | Ras signaling pathway | 3.8127E-09 | 9.72E-07 |
| KEGG:04390 | Hippo signaling pathway | 5.5521E-09 | 1.41E-06 |
| KEGG:04012 | ErbB signaling pathway | 6.1100E-09 | 1.55E-06 |
| KEGG:04550 | Signaling pathways regulating pluripotency of stem cells | 8.8309E-09 | 2.23E-06 |
| KEGG:04261 | Adrenergic signaling in cardiomyocytes | 9.6232E-09 | 2.42E-06 |
| KEGG:05202 | Transcriptional mis regulation in cancer | 1.1172E-08 | 2.79E-06 |
| KEGG:04933 | AGE-RAGE signaling pathway in diabetic complications | 1.9261E-08 | 4.80E-06 |
| KEGG:04068 | FoxO signaling pathway | 3.4018E-08 | 8.44E-06 |
| KEGG:05211 | Renal cell carcinoma | 3.9351E-08 | 9.72E-06 |
| KEGG:05322 | Systemic lupus erythematosus | 7.2086E-08 | 1.77E-05 |
| KEGG:04310 | Wnt signaling pathway | 1.2732E-07 | 3.12E-05 |
| KEGG:05215 | Prostate cancer | 1.3807E-07 | 3.37E-05 |
| KEGG:04912 | GnRH signaling pathway | 1.7236E-07 | 4.19E-05 |
| KEGG:04919 | Thyroid hormone signaling pathway | 2.5789E-07 | 6.24E-05 |
| KEGG:04140 | Autophagy | 3.9575E-07 | 9.54E-05 |
| KEGG:04072 | Phospholipase D signaling pathway | 4.6444E-07 | 1.11E-04 |
| KEGG:04916 | Melanogenesis | 6.4233E-07 | 1.54E-04 |
| KEGG:05212 | Pancreatic cancer | 1.0021E-06 | 2.39E-04 |
| KEGG:05224 | Breast cancer | 1.0685E-06 | 2.53E-04 |
| KEGG:05222 | Small cell lung cancer | 1.1478E-06 | 2.71E-04 |
| KEGG:04150 | mTOR signaling pathway | 1.3174E-06 | 3.10E-04 |
| KEGG:00982 | Drug metabolism | 1.6166E-06 | 3.78E-04 |
| KEGG:04151 | PI3K-Akt signaling pathway | 2.0545E-06 | 4.79E-04 |
| KEGG:04924 | Renin secretion | 2.2950E-06 | 5.32E-04 |
| KEGG:04728 | Dopaminergic synapse | 3.3893E-06 | 7.83E-04 |
| KEGG:04723 | Retrograde endocannabinoid signaling | 3.7482E-06 | 8.62E-04 |
| KEGG:05220 | Chronic myeloid leukemia | 3.9845E-06 | 9.12E-04 |
| KEGG:05214 | Glioma | 4.0173E-06 | 9.16E-04 |
| KEGG:04152 | AMPK signaling pathway | 4.1502E-06 | 9.42E-04 |
| KEGG:04022 | cGMP-PKG signaling pathway | 4.6007E-06 | 0.001039761 |
| KEGG:05218 | Melanoma | 5.1152E-06 | 0.001150924 |
| KEGG:04520 | Adherens junction | 8.2162E-06 | 0.001840426 |
| KEGG:05223 | Non-small cell lung cancer | 9.4755E-06 | 0.002113037 |
| KEGG:04371 | Apelin signaling pathway | 1.0238E-05 | 0.002272817 |
| KEGG:04211 | Longevity regulating pathway | 1.0249E-05 | 0.002265046 |
| KEGG:04931 | Insulin resistance | 1.3086E-05 | 0.002878899 |
| KEGG:04720 | Long-term potentiation | 1.5718E-05 | 0.003442314 |
| KEGG:04810 | Regulation of actin cytoskeleton | 1.5781E-05 | 0.003440181 |
| KEGG:04115 | p53 signaling pathway | 1.8471E-05 | 0.004008223 |
| KEGG:03008 | Ribosome biogenesis in eukaryotes | 1.9325E-05 | 0.00417424 |
| KEGG:04921 | Oxytocin signaling pathway | 1.9365E-05 | 0.004163463 |
| KEGG:04660 | T cell receptor signaling pathway | 2.2232E-05 | 0.004757668 |
| KEGG:00590 | Arachidonic acid metabolism | 2.2369E-05 | 0.004764506 |
| KEGG:04024 | cAMP signaling pathway | 2.3697E-05 | 0.005023805 |

**Table S5: Sample used in the study**

| **Technique** | **Samples used** | |
| --- | --- | --- |
| Sequencing | Pooled enriched CESCs from 2 donors  Total cells:2046 | Pooled CCECs from 2 donors  Total cells:8.55×10^5^ |
| qPCR | CESCs (n=3 pools)  Pool 1: 11 pairs  Pool 2: 11 pairs  Pool 3: 11 pairs | CCECs (n=3) |
|  | LBECs (n=3 pools)  Pool 1: 7 pairs  Pool 2: 7 pairs  Pool 3: 6 pairs | CCECs (n=3 pools)  Pool 1: 7 pairs  Pool 2: 7 pairs  Pool 3: 6 pairs |
